# A pediatric disease to keep in mind: diagnostic tools and management of bronchiectasis in pediatric age

**DOI:** 10.1186/s13052-017-0434-0

**Published:** 2017-12-29

**Authors:** Marcella Gallucci, Emanuela di Palmo, Luca Bertelli, Federica Camela, Giampaolo Ricci, Andrea Pession

**Affiliations:** Pediatric Unit, Department of Medical and Surgical Sciences, S. Orsola – Malpighi Hospital, University of Bologna, Via Massarenti 11, 40138 Bologna, Italy

**Keywords:** Children bronchiectasis; chronic wet cough, HRCT, MRI, Antibiotics, Azithromycin, Airway clearance techniques

## Abstract

Bronchiectasis in pediatric age is a heterogeneous disease associated with significant morbidity.

The most common medical conditions leading to bronchial damage are previous pneumonia and recurrent lower airway infections followed by underlying diseases such as immune-deficiencies, congenital airway defects, recurrent aspirations and mucociliary clearance disorders.

The most frequent symptom is chronic wet cough. The introduction of high-resolution computed tomography (HRCT) has improved the time of diagnosis allowing earlier treatment.

However, the term “bronchiectasis” in pediatric age should be used with caution, since some lesions highlighted with HRCT may improve or regress. The use of chest magnetic resonance imaging (MRI) as a radiation-free technique for the assessment and follow-up of lung abnormalities in non-Cystic Fibrosis chronic lung disease is promising.

Non-Cystic Fibrosis Bronchiectasis management needs a multi-disciplinary team. Antibiotics and airway clearance techniques (ACT) represent the pillars of treatment even though guidelines in children are lacking. The Azithromycin thanks to its antinflammatory and direct antimicrobial effect could be a new strategy to prevent exacerbations.

## Background

Non-cystic fibrosis bronchiectasis (NCFB) is a chronic pulmonary disease characterized by a progressive and often irreversible bronchial dilatation, caused by structural changes in the bronchial wall and chronic airway inflammation.

The actual incidence is unknown but the prevalence has increased in developing countries and among lowest socioeconomic classes where the disease often shows an infectious etiology.

The highest NCFB prevalence is among children of indigenous populations, such as Australian aborigines, New Zealand Maoris, Alaskan natives and Pacific Islander children [1].

In this respect, two New Zealand studies over the period 1998–2002, one reporting national cases of chronic suppurative lung disease (CSLD) and other CSLD hospital admissions in Auckland, showed a considerable disproportion between the average annual incidence among Maori and Pacific Islander children (respectively 4.8–7.9/100,000 and 17.8–18.3/100,000) compared to European children (1.5/100,000). This difference seems to be directly related to the poor condition in which these populations live. The incidence among European populations is estimated to be around 0.2 / 100,000 in UK and 2,3/100,000 in Ireland [2].

Nevertheless, although NCFB is considered a disease of poverty, data on prevalence over the past two decades show that also in industrialized country this diagnosis is no longer so rare, probably thanks to advances in diagnostic techniques such as high-resolution computerized tomography (HRCT) [3].

The pathogenesis of NCFB is not fully known yet but seems to involve factors related to the host and microorganisms such as defective mucociliary clearance and persistent inflammation associated with bronchial persistent infection.

Historically bronchiectasis are defined as “cylindrical, varicose or cystic” by bronchographic imaging and these characterizations respectively seems to be correlated with an increased degree of severity [4]. In a systematic review involving 989 children, Brower et al. showed that 63% had an underlying disease [5].

Excluding Cystic fibrosis, previous pneumonia and recurrent lower airway infections are the most common causes of pediatric bronchiectasis; others risk factors includes primary immune deficiencies, primary ciliary dyskinesia (PCD), foreign body aspiration and structural airways abnormalities such as bronchomalacia and congenital tracheo-bronchomegaly (Table 1) [6].

**Table 1:** Most common causes and associations of NCFB in pediatric age

CFTR mutations	Cystic fibrosis classic and atypical
Dysregulated immune function	Primary immune-deficiency
	X-linked Agammaglobulinemia
	Common variable immunodeficiency (CVID)
	Hyper–immunoglobulin E syndrome or STAT3^a^ deficiency
Primary Ciliary Dyskinesia (PCD)	PCD
	Kartagener Syndrome
Post-Infectious disease	Bacterial Pneumonia
	Recurrent respiratory tract infections
	Protracted bacterial bronchitis (PBB)
Structural congenital malformations	Congenital tracheo-broncomegaly: Mounier-Kuhn syndrome William-Campbell syndrome
	Congenital lobar emphysema
	Bronchomalacia
Bronchial obstruction	Inhaled foreign body
	Tumor
	Mycobacterium
Associated conditions	Recurrent aspiration secondary to neuromuscular diseases
	Tracheo-esophageal fistula or gastro-esophageal reflux
Others	Autoimmune diseases Inflammatory bowel disease Rheumatoid arthritis Coeliac disease
	Clinical syndromes Marfan syndrome Usher syndrome Yellow nail lymphedema syndrome Young’s syndrome

^a^signal transducer and activator of transcription 3

Children with Protracted Bacterial Bronchitis (PBB) exhibit a condition of persistent endobronchial infection and neutrophilic inflammation, known risk factors for the onset of NCFB. These factors have led to the hypothesis according to which the PBB and NCFB represent different stages of a single disorder [7].

Patients with PCD have a congenital abnormality of ciliary function that causes impairment of mucociliary clearance and chronic respiratory tract infections often beginning in the first years of life. A history of neonatal respiratory distress without prematurity and recurrent middle ear problems are both very evocative of diagnosis; chronic rhino-sinusitis and bronchiectasis may be observed among older children [8].

Bronchiectasis can also complicate primary and secondary immune-deficiencies. Defective immune function is associated with repeated, chronic and severe lung infections leading to recurrent episodes of airways inflammation, repair and ultimately structural damage.

Most frequent immune disorders include defective production or function of all immunoglobulin classes, individual classes or subclasses and defects of specific antibody production such as bacterial capsular polysaccharide [9].

Aspiration of gastrointestinal contents has been proved as a cause of bronchiectasis in both children and adults even though there are no case control studies of gastro-esophageal reflux as a risk factor for bronchiectasis in children [10]. Conversely, it is well known that children with neuromuscular diseases and/or degenerative pathologies often have a poor cough reflection efficiency that makes them more susceptible to recurrent aspiration of both gastric content and respiratory pathogens that cause recurrent endobronchial infections and subsequent bronchiectasis.

## Clinical presentation

NCFB in children usually exhibits chronic respiratory symptoms.

Chronic wet cough is the commonest finding and may be present for many years before reaching a diagnosis. The likelihood of bronchiectasis increases if these symptoms are accompanied by exertional dyspnea, recurrent infections of the lower airways, hemoptysis and chest wall deformities [10]. Recurrent wheezing, dyspnea and growth failure are additional symptoms that may be associated with NCFB in pediatric age (Table 2) [3, 11, 12].

Repeated episodes of pulmonary exacerbations are also associated with progressive decline in lung function and reduced quality of life.

At present, there is no validated definitions of “exacerbation” in children with NCFB. Exacerbation criteria used in adults (increased cough, increased sputum volume and worsening of purulence) are less useful in children who are often unable to expectorate [13].

In a recent retrospective study involving 115 children with NCFB, Kapur et al. highlighted that an increase in cough frequency and modifications in its features, an increase of auscultation findings of crepitations and wheezing were useful clinical indicators of exacerbation [14].

Infections are common cause of exacerbations whose triggers can be either respiratory viruses or bacteria, hence an accurate etiological diagnosis might reduce the use of antibiotic therapy and the risk of resistance [15].

## Diagnostic tools

No standardized method for classifying bronchiectasis has been defined in pediatric age, even though clinical, radiological and microbiological findings are usually used to quantify the disease severity [16].

A radiological diagnosis of bronchiectasis in the child should be done with caution since a radiological regression of lesions has been observed after treatment [11, 17]. Chest radiography has a poor diagnostic value and therefore a compatible clinical history should prompt additional investigation. Nevertheless, chest x-ray is usually the first imaging test used in the clinical suspect of NCFB and some studies have suggested that it is rarely completely negative in cases of clinically relevant lesions. For this reason, according to our opinion, performing a chest x-ray may be justified at the beginning of the diagnostic path [18].

**Fig. 1:**
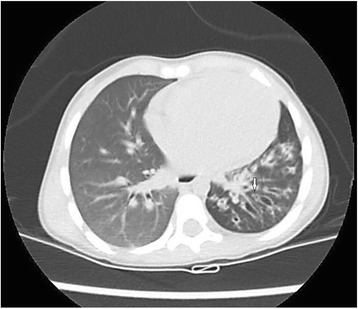
HRCT of a 3-year-old child showing several cylindrical bronchiectasis of the lower left lobe. The arrow indicates the classic “signet ring sign”

High-resolution computerized tomography (HRCT) is the gold standard for the diagnosis of NCFB. The commonest HRCT findings include bronchial dilatation, bronchial wall thickening, lack of normal bronchial tapering, any bronchi with an internal diameter greater than the diameter of the accompanying pulmonary artery (signet ring sign) and bronchi visible closer than two centimeters to the pleural surface (Table 2). In children a broncho-arterial ratio > 0,8 is considered typical of bronchiectasis (Fig. 1) [19].

Mosaic perfusion defects and air trapping on expiration can also be observed.

Although a tendency to certain distributions has been described in groups of patients with specific conditions, the localization and pattern of abnormalities on HRCT is not necessarily linked to the etiology [6, 20].

Several radiographic scores such as Bhalla, Nathanson, Reiff, and Webb’s scores, use combination of CT findings as markers of disease extension and severity [21–24].

Bhalla score is a commonly accepted score for children. It includes several items such as:

a) severity of bronchiectasis; b) peribronchial thickening; c) extent of bronchiectasis (number of lung segments); d) extent of mucus plugs; e) abscesses or sacculations; f) generalities of the bronchial division involved (bronchiectasis/plug); g) number of bubbles; h) emphysema (number of lung segments); i) collapse/consolidation [24, 25].

According to Goyal and colleagues, HRCT should be considered in every child with a chronic wet cough persisting after 4 week of oral antibiotics [26]. Nevertheless, false negative results in children may be caused by motion artifacts or poor collaboration, therefore an evocative clinical history should suggest diagnosis even in absence of the above-mentioned radiological criteria.

In recent years, chest magnetic resonance imaging (MRI) has been proposed as a radiation-free technique for the assessment and follow-up of several chest disorders.

Montella et coll. in a recent prospective study have shown that high-field 3.0-T MRI had a good correlation with HRCT in identifying bronchiectasis and the commonest alterations related to non-CF lung disease (mucous plugging, peribronchial wall thickening, consolidation, bullae, abscesses and emphysema) [20, 27].

Similarly, Maglione et al. in their recent comparative study of PCD and FC highlighted an excellent agreement between MRI and CT for most abnormality parameters in both diseases [28].

Moreover, for some findings such as mucus plugging, MRI may be more helpful and informative than HRCT, thanks to the high protonic density of mucous, which is more easily distinguished from bronchial wall thickening. Consequently, MRI may be more sensitive in detecting early airway wall changes and mucus retention that may precede more serious structural damage in the larger airways.

In addition, MRI may identify some functional aspects of lung tissue such as lung perfusion or ventilation and since it does not expose the patient to ionizing radiation, it may be particularly useful in the long-term monitoring of lung diseases [29]. Nevertheless, MRI has less sensitivity in detection of peripheral bronchiectasis without bronchial wall thickening or nodules smaller than 5 mm.

Spirometry can be useful to give additional and functional information on disease severity in older children and can reveal either an obstructive or mixed obstructive/restrictive airflow pattern.

Several evidences indicate that reduced FEV1 is associated with more severe disease and severity of structural abnormality highlighted with HRCT, but it does not always correlate with the clinical course and it has a poor sensitivity in detecting exacerbations [30].

Furthermore, spirometry performed during radiological examinations (chest CT and MRI) can improve image quality and the detection of bronchiectasis so it is useful in order to standardize lung volumes [31].

Other tests such as measurement of static lung volumes and the 6-min walk test may be used to assess functional impairment. Exercise testing may be more informative than static spirometry in assessment of lung function, especially in patients whose performance or symptoms do not correlate with spirometric findings [32].

In the last years, there has been a growing interest in the multiple breath inert gas washout (MBW), a technique used to quantify ventilation heterogeneity, which is considered an early sign of chronic obstructive lung diseases such as asthma and cystic fibrosis. Airway narrowing due to mucus retention, inflammation and airway wall structural damage causes inhomogeneity of lung ventilation. This inhomogeneity compromises the gas mixing efficiency of the lung and can be measured by following the washout of an inert gas during tidal breathing. In particular, lung clearance index (LCI) is a measure of lung physiology derived from multiple breath washout tests and is defined as the cumulative expired volume (CEV) at the point where end-tidal inert gas concentration has fallen to 1/40th of the starting concentration, divided by the functional residual capacity (FRC).

Moreover, since only tidal breathing is required, LCI is simple and reproducible hence it is ideal for use in children [33].

Recently, some study have showed that in non-CF bronchiectasis LCI correlates with CT severity score and with spirometric markers of airway obstruction. In addition, these parameters are abnormally increased in a significant percentage of non-CF bronchiectasis patients with a normal FEV1. These findings suggest that LCI may be a potential marker of disease severity and a useful index of airflow obstruction, although further studies are required to validate this hypothesis [34].

Bronchoscopy is helpful to identify underlying structural anomalies and to obtain lower airway secretions in children unable to expectorate [35]. A retrospective study involving 93 children with wet cough showed that flexible bronchoscopy with bronchoalveolar lavage (BAL) is more sensitive than HRCT in detecting airway abnormalities therefore both these tools should be complementary to confirm the diagnosis and to assess the disease severity [36].

Finally, bronchiectasis may be considered as the result of an altered balance between degradation and deposition of the extracellular matrix resulting from a modification of the relationship between metalloproteases and their tissue inhibitors. For these reason, the detection of metalloproteases (MMP-9) in exhaled breath condensate could be a useful marker of airway injury in patients with NCFB [37].

**Table 2:** Most common findings of NCFB in children

Symptoms	Clinical indicators of exacerbation	HRCT findings	Most common isolated bacteria
Chronic wet cough	Increase in frequency of cough	Bronchial dilatation	Haemophilus influenzae
Recurrent chest infections	Change of cough character	Bronchial wall thickening	Streptococcus pneumonia
Exertional dyspnea	Increase in crepitations	Lack of normal bronchial tapering	Moraxella catarrhalis
Recurrent wheezing	Wheeze	Signet ring sign	*Staphylococcus aureus*
Delayed growth		Bronchi visible closer than 2 cm to the pleural surface	Pseudomonas aeruginosa (in older children)
Chest wall deformity		Broncho-arterial ratio > 0,8	

## Management

### Antibiotics

NCFB requires multidisciplinary management in order to control symptoms, reduce exacerbations and preserve lung function.

Although only one organ is concerned, the disease can be complicated by a reduction in statural weight gain and poor overall quality of life.

Redding and coll. in a prospective study monitoring 93 children with NCFB for 3 years, reported that more than two exacerbations occurred in the majority of cases (74%) and a complete medical care was associated with a reduction of exacerbation rate [38].

**Fig. 2:**
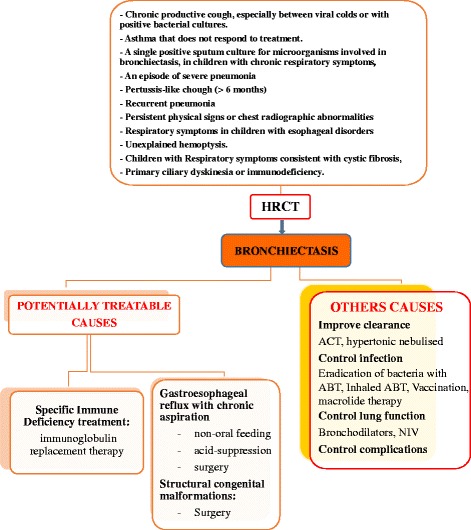
Management of NCFB

Antibiotics and airway clearance techniques (ACTs) represent the milestones of NCFB management although the guidelines in children are only a few (Fig. 2).

Antibiotics should be used in the treatment of acute exacerbation or as prophylaxis treatment to reduce the frequency of acute events. The use and choice of antibiotics should be guided by exacerbations severity, airway microbiology results (sputum culture) and whenever possible by BAL culture. The most commonly isolated bacteria in children are non-encapsulate *Haemophilus influenzae, Streptococcus pneumonia, Moraxella catarrhalis* and *Staphylococcus aureus*. *Pseudomonas aeruginosa* is rarely found in the childhood and it is more often detected in older children or associated with underlying diseases with increased lung damage [39, 40]. Non-typeable H. Influenzae is the major pathogen detected in children of all ages affected by NCFB. This microorganism is able to form a biofilm on the airway epithelia that damages cilia and impairs the bronchial clearance mechanism [41].

In our opinion, bacteria involved in exacerbations should always be identified in order to use a targeted antibiotic therapy that minimizes the future risk of resistance.

Sputum sample culture or nasopharyngeal aspirates (in children who are unable to expectorate) should be obtained before starting antibiotics. A pharyngeal swab after coughing may be a surrogate of the lower respiratory tract pathogens in very young children and it is sometimes preferred in some older children [42].

To date, despite the advances in the management of many pediatric diseases antibiotic therapy remains one of the most effective tool for the treatment of pediatric NCFB.

Prolonged cycles of antibiotics (very often more than 4 weeks) are often necessary to eradicate some respiratory pathogens responsible for exacerbation [39].

A recent review involving 15 studies (925 participants), showed a significantly reduction in the rate of exacerbations and hospitalization in children and adults with NCFB who had been treated with prolonged antibiotics (four or more weeks). However, the risk of emerging drug resistance was increased more than threefold, therefore an accurate patient selection is essential [43].

Unfortunately, there are currently no randomized controlled trials (RCTs) evaluating the efficacy of inhaled antibiotics in children with NCFB, even though this option in adult patients has been largely used and showed several benefits. The rationale for using inhaled antibiotics in lung diseases lies in the possibility to administer a relatively high dose of drugs directly to the site of disease reducing systemic absorption and related toxicity.

A systematic review conducted by Brodt et al. comprising 12 trials with 1264 adults with clinically stable NCFB and chronic bronchial infection, showed that inhaled antibiotics were more effective than placebo in reducing sputum bacterial load and the risk of acute exacerbations [44].

According to current BTS guidelines, nebulized antibiotics should be considered in children who experience recurrent exacerbations (or deteriorating lesions) unaffected by prolonged oral antibiotic or if colonized with Pseudomonas Aeruginosa [45].

Nebulized gentamicin in children with NCFB seems to be well tolerated and produce satisfactory drug level in the sputum. Nebulized tobramycin was mainly used in children with CF and demonstrated good efficacy in reducing the concentration of Pseudomonas and improving the FEV1 [46]. However, there are a few data in NCFB and in any case, the choice of the nebulized antibiotic should be guided by culture results.

In the recent years, there is an increased interest in the use of macrolides for the treatment of NCFB, mainly due to their antinflammatory effects and their well-established direct antimicrobial effect on Gram-positive cocci and atypical pathogens but also for the properties to decrease mucus production.

Two meta-analyses that included seven studies evaluating various macrolides (azithromycin, erythromycin, roxithromycin) reported a significant reduction in the frequency of exacerbations compared with placebo [39, 43].

Similarly, the EMBRACE trial (Effectiveness of Macrolides in patients with Bronchiectasis using Azithromycin to Control Exacerbations) involving three centers in New Zealand, showed that the use of Azithromycin three times a week for 6 months in adult patients with NCFB has resulted in a significant reduction in event-based exacerbations and increase of the time of the first event-based exacerbation compared with placebo [47].

Despite these encouraging evidences, the risk of drug resistances should not be underestimated. In that regard, in BLESS study (Bronchiectasis and Low-dose Erythromycin Study) Rogers and colleagues reported changes in microbiota composition of patients with NCFB (determined by 16S rRNA gene sequencing of sputum samples from 86 participants) after 48 weeks of low-dose erythromycin. Moreover, long-term erythromycin treatment seems to induce replacement of H. influenzae with more macrolide-tolerant pathogens such as *P. aeruginosa*. These evidences suggest that chronic macrolide therapy should be used with caution in subjects with NCFB but without *P. aeruginosa* infection [48].

### Airway clearance techniques

Airway clearance techniques (ACTs) are usually recommended in children with bronchiectasis even though evidences supporting this practice are lacking and mainly derives from study on Cystic Fibrosis.

In the childhood, the family compliance may significantly influence the adherence to ACT and the non-adherence is the primary cause of treatment failure in chronic pediatric lung conditions. Other factors to consider when choosing ACT are child’s age, level of collaboration and maturity.

Lee et colleagues showed that ACTs seemed to be safe for adults and children with stable bronchiectasis leading to improvements in sputum expectoration, selected measures of lung function, patient symptoms and HRQoL (health related quality of life) [49].

Different types of ACTs include: active cycle of breathing techniques, autogenic drainage, forced expiration techniques, postural drainage, oscillating positive expiratory pressure, high-frequency chest wall oscillation, and physical exercise. This latter is highly recommended for all ages in the management of bronchiectasis and chronic suppurative lung disease.

Furthermore, the application of positive expiratory devices and high-frequency chest wall oscillation showed an improvement of FEV1 as indices of lung function. However, all the studies were conducted in individuals in a stable clinical state, so the effects of these techniques during an acute exacerbation remain unclear and further studies are necessary, especially in children [50, 51].

Finally, the use of both beta2 agonists and hypertonic solutions before performing airway clearance techniques are all considered useful strategies for improving clearance of bronchial secretions in NCFB.

### Mucolytic agents

The mucolytic agents could theoretically be useful in the management of bronchiectasis due to their ability to reduce the viscosity of the mucus and thus make it easier to expectorate. Among these drugs, recombinant human DNase, acts in degrading DNA released by neutrophils in the infection site.

A recent Cochrane review involved four trials with 528 adult participants, concluded that little evidence is available to recommend the routine use of mucolytic agents in bronchiectasis [52].

In 2015 Welsh et al. in an “overview of Cochrane systematic reviews” regarding the various treatments currently available for non-cystic fibrosis bronchiectasis, reported that RhDNase seems to be associated with an increase in exacerbations, although three reviews only involved pediatric population.

Current BTS guidelines conclude that due to a potential worsening of pulmonary function, RhDNase should not be used in management of bronchiectasis in pediatric patients [53].

Two reviews reported beneficial effects for other agents such as hypertonic saline (exacerbations), erdosteine (FEV1) and mannitol (QoL) [52].

About this last one, a recent RCT showed that the use of mannitol 400 mg inhaled twice daily for 12 months in adult patients with bronchiectasis seems to improve the quality of life and increase the time to the first exacerbation even though it did not reduce exacerbation rates [54, 55].

The role of hypertonic saline in the management of NCFB is still debated. Although the recent Cochrane systematic reviews of Welsh et al. concluded that there is inconclusive evidence on the use of nebulized hypertonic for reducing exacerbation, several studies suggested that in concentrations of 3–14%, hypertonic saline could improve tracheobronchial clearance.

A probable mechanism of action consists in the reduction of sputum viscosity by inducing liquid flux from the epithelium into the mucus so that the mucus is cleared more easily by the cilia.

Kellet and coll. Demonstrated that nebulized hypertonic saline significantly reduced sputum viscosity and can be used safely and effectively as an adjunct to physiotherapy in patients with stable bronchiectasis [56, 57].

### Combined ICS/LABA therapy

Several studies have reported the presence of a certain degree of bronchial hyper-responsiveness and chronic airflow bronchial obstruction in subjects with bronchiectasis. For this reason, inhaled corticosteroids (ICS) therapy may potentially improve several bronchial inflammatory parameters and clinical symptoms, even though high doses with increased risk of side effects are often required [58].

The use of combined therapy with long-acting beta2-agonist (LABA) and ICS administered together in a single device showed a synergistic effect in decreasing bronchial inflammation.

A Cochrane’s systematic reviews of all randomized controlled trials on the use of combined ICS and LABA compared to a control (placebo, no treatment, ICS as monotherapy) in children and adults with bronchiectasis, concluded that in adults combined therapy was partially more effective than high-dose ICS in improving some clinical symptoms like cough free-day and dyspnea. However, no significant improvement in the rate of hospitalization and no change in lung function indices have emerged. Moreover, no data are provided in children with bronchiectasis in a stable or acute state [59].

A single-center observational study performed by Wei et al. involving 120 adult patients with NCFB, reported that subjects who received salmeterol-fluticasone combined therapy versus routine therapy showed significant improvement in HRQoL and rates of exacerbations, without risk of severe adverse events [60].

Previously, Martinez-Garcia also reported in a 12-month randomized and double-blind trial including 40 patients with NCFB, that inhaled medium-dose of formoterol-budesonide combined therapy was more effective and safe compared with high-dose of budesonide in improving clinical symptoms such as dyspnea, cough and wheeze [61].

Despite these encouraging findings, there is a lack of safety data about the combined ICS/LABA therapy in children, therefore more robust evidence is needed to recommend its routinely use in NCFB.

### Surgical treatment

Finally, surgical treatment such as segmentectomy and lobectomy may be reserved for patients with localized bronchiectasis with persistent symptoms, recurrent infections despite maximal therapy and hemoptysis. Nevertheless, further studies are needed before surgery can be recommended as a safe treatment for NCFB. Factors that seem to be relative contraindications for surgical resection of bronchiectasis include non-cylindrical disease, persistent infection of Pseudomonas documented by sputum culture, residual disease after resection and non-localized disease [62–65].

## Conclusions

NCFB is an emerging pediatric disease whose prevalence is probably underestimated worldwide.

The most common causes include infections, immunodeficiency, recurrent aspiration and primary ciliary dyskinesia. An early diagnosis is essential to prevent a progressive decline in lung function and recurring exacerbations that are inevitably associated to a poorer quality of life.

An etiological diagnosis is also essential in order to improve the course of the disease by using specific therapy (e.g. specific replacement therapy in some immunodeficiency cases or anti-acid treatment in recurrent aspirations with gastroesophageal reflux) (Fig. 2).

The use of HRCT represents the gold standard to confirm the diagnosis although magnetic resonance imaging (MRI) could be a promising radiation-free technique to identify NCFB and to follow its evolution over time.

Actually, the pillars of treatment are represented by targeted antibiotic therapy in combination with airway clearance techniques. Prolonged antibiotics are often necessary to decrease the bacterial load, therefore the risk of emerging drug resistances should not be overlooked. Inhaled antibiotics, inhaled hypertonic and inhaled mannitol can be useful instruments in improving the quality of life.

Finally, Azithromycin and other macrolides seem to reduce the frequency of event-based exacerbations compared with placebo, therefore it may be a precious option in NCFB management.

## Abbreviations

ACT: Airway clearance techniques
BAL: Bronchoalveolar lavage
HRCT: High-resolution computerized tomography
MRI: Magnetic resonance imaging
NCFB: Non-cystic fibrosis bronchiectasis
PCD: Primary ciliary dyskinesia
